# Extracorporeal life support and continuous renal replacement therapy in a patient with Enterovirus A71 associated cardiopulmonary failure: A case report

**DOI:** 10.1097/MD.0000000000036797

**Published:** 2024-01-05

**Authors:** Nguyen Trung Bao, Vo Thanh Luan, Bui Thanh Liem, Vo Hoang Thien Nhu, Do Chau Viet, Trinh Huu Tung, Sakib Burza, Nguyen Tat Thanh

**Affiliations:** a Department of Infectious Diseases, Children Hospital No.2, Ho Chi Minh City, Vietnam; b Faculty of Pediatrics, University of Medicine and Pharmacy, Ho Chi Minh City, Vietnam; c University of Medical Center, Ho Chi Minh City, Vietnam; d London School of Hygiene and Tropical Medicine, London, United Kingdom; e Health in Harmony, London, United Kingdom; f Woolcock Institute of Medical Research, Ho Chi Minh City, Vietnam.

**Keywords:** CRRT, enterovirus A71, extracorporeal membrane oxygenation, hand foot and mouth disease, Vietnam

## Abstract

**Rationale:**

Hand-foot-mouth disease (HFMD) caused by Enterovirus A71, complicated by cardiopulmonary failure, is associated with a high mortality rate despite intensive treatment. To date, there is a paucity of clinical management data, regarding the use of extracorporeal life support (VA-ECMO) for Enterovirus-A71 associated cardiopulmonary failure reported.

**Patient concerns:**

The patient in this study presented with severe HFMD complicated by cardiopulmonary failure, polymorphic ventricular tachycardia, and cardiac arrest.

**Diagnoses:**

Clinical presentations, laboratory data, and polymerase chain reaction (PCR) results from rectal swabs were used to confirm the diagnosis of severe HFMD caused by Enterovirus A71.

**Interventions:**

The patient was managed with chest compression and an automatic external defibrillator, mechanical ventilation, intravenous immunoglobulin (IVIG), continuous renal replacement therapy (CRRT) and inotrope (milrinone). The patient did not respond to these interventions and subsequently required further management with VA-ECMO.

**Outcomes:**

The patient achieved a favorable outcomes.

**Lessons:**

Our study highlights that extracorporeal membrane oxygenation and CRRT can enhance the survival outcomes of patients with severe HFMD with cardiopulmonary failure complications. Furthermore, we propose specific indications for the initiation of VA-ECMO.

## 1. Introduction

Hand foot and mouth disease (HFMD) is primarily caused by the Enterovirus family, including Enterovirus A71 and Coxsackievirus A6, A10, and A16.^[1]^ HFMD is prevalent in many parts of the world but is more concentrated in the Western Pacific and parts of Asia, with large outbreaks.^[2]^ In Vietnam, there have been 2 major outbreaks in 2011 to 2012 (>200,000 admissions and > 200 deaths) and in 2018 (>130,000 admissions and 17 deaths) reported.^[1]^ In most cases, HFMD is mild and self-limiting, with a small proportion of patients progressing to severe HFMD, which can lead to life-threatening conditions such as central nervous system (CNS) involvement, pulmonary edema, and cardiopulmonary failure.^[2]^ Severe HFMD complicated by cardiopulmonary failure accounts for a high mortality rate, ranging from 26% to 70%.^[3,4]^ Importantly, Enterovirus A71 is primarily responsible for most patients with severe HFMD in Vietnam.^[5]^ To date, there are no specific therapies for severe HFMD, particularly those caused by Enterovirus A71. Conventional therapies for severe HFMD with CNS and cardiopulmonary complications include mechanical ventilation, intravenous immunoglobulin, vasopressors, and inotropes.^[2]^ Notably, continuous renal replacement therapy (CRRT) and extracorporeal membrane oxygenation (VA-ECMO) are considered rescue treatments for patients with severe HFMD who experience cardiopulmonary failure and do not respond to conventional therapies.^[2,3,6]^

To date, there are limited clinical data on the use of VA-ECMO resuscitation in children with severe HFMD. In 2010, Jan et al reported a case series of 13 children with Enterovirus-A71-associated acute cardiopulmonary failure who received VA-ECMO. Among these, 10/13 patients were weaned from VA-ECMO, 4 later died, and another survived with critical neurological sequelae.^[3]^ Another study by Jan et al in 2021 involved 17 children with severe HFMD complicated by Takotsubo syndrome due to a catecholamine storm; 15 patients could be weaned from VA-ECMO intervention; however, one patient died soon after, and the other 3 patients experienced critical neurological sequelae.^[4]^ A meta-analysis demonstrated a high rate of cerebral sequelae in 38.5% of severe HFMD patients with cardiopulmonary failure.^[7]^ Therefore, VA-ECMO interventions should be carefully considered in resource-limited countries. Here, we describe the first patient in Vietnam with severe HMFD complicated by cardiopulmonary failure, refractory ventricular tachycardia arrhythmia, and cardiac arrest who was successfully treated with combined CRRT and VA-ECMO at our tertiary pediatric hospital.

## 2. Case presentation

**Figure 1.**
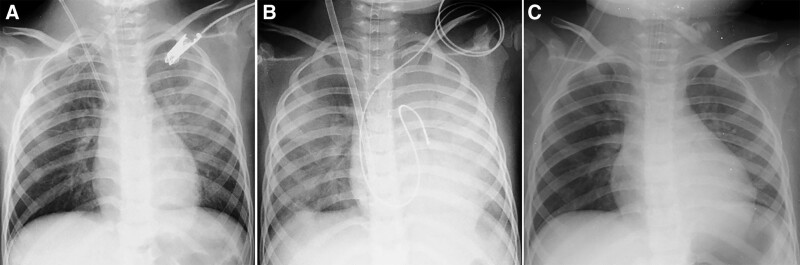
Chest radiography showed substantial bilateral pulmonary interstitial congestion on PICU admission (A) at the start of extracorporeal membrane oxygenation (VA-ECMO) (B), and marked improvement after VA-ECMO (C).

**Figure 2.**
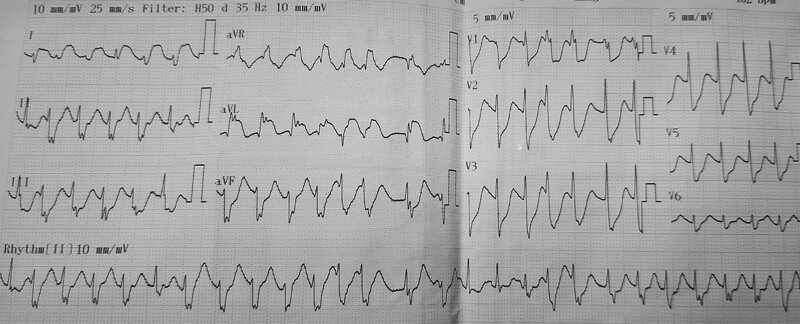
Electrocardiography revealed refractory ventricular tachycardia with 200 beats per minute.

A 5-year-old female from Ho Chi Minh City presented in July 2023 with a history of 3 days of high fever and recent onset of breathing difficulty. She had no underlying diseases until hospital admission. Her main symptoms were persistent high fever, vomiting, malaise, markedly startling jerks, and weakness of the upper limbs. Upon admission to the pediatric intensive care unit (PICU), she was alert but lethargic, had mottled skin and pale mucosa, cool extremities, respiratory rate of 70 breaths per minute, increased heart rate of 198 beats per minute (bpm), blood pressure reading of 128/78 mm Hg, mouth ulcers, undetected skin rash or vesicles, tachycardia and moist rales, soft abdomen, muscle tone of 5/5 in the lower limbs, and 3/5 in her right arm and 5/5 in her left arm. Laboratory results on admission revealed elevated white blood cell counts 18 × 10^9^/L, normal values for hematocrit, platelet count, electrolytes and coagulation profiles, blood glucose 200 mg%, aspartate transaminase (AST) 49 U/L and alanine transaminase (ALT) 15 U/L, serum lactate 5.8 mmol/L, serum creatinine 47 µmol/L, procalcitonin 0.9 ng/mL, cardiac Troponin I 7.36 ng/L and positive Enterovirus-A71 IgM ELISA-based tests. Arterial blood gases analysis revealed pH 7.38, pCO2 11 mm Hg; pO2 115 mm Hg; base excess (BE) −18.4 and HCO3 6.2 mEq/L (Table 1). Chest radiography revealed bilateral pulmonary interstitial congestion (Fig. 1A). She was diagnosed with severe hand-foot mouth disease caused by Enterovirus A71. She was initially managed with mechanical ventilation, intravenous immunoglobulin, inotrope (milrinone), and continuous renal replacement therapy (CRRT). Four hours after initiating CRRT, her clinical status progressively deteriorated, and she further developed polymorphic ventricular tachycardia with 200 beats per minute, as observed on electrocardiogram (ECG) (Fig. 2). Physical examination revealed a blood pressure of 70/35 mm Hg, capillary refill time (CRT) > 3 seconds, response to pain stimulus, pupillary diameter of 2 mm, and positive light reflex. A point-of-care cardiac ultrasound at the bedside revealed a hypodynamic heart, no signs of cardiac effusion, and a low ejection fraction (EF) of 30%. Monitored laboratory tests showed huge increases in cardiac Troponin I (>50 ng/L) and serum lactate > 13.4 mmol/L, and arterial blood gases indicating pH 7.21, pCO2 21 mm Hg, pO2 185 mm Hg, BE −19.5 and HCO3 7.4 mEq/L. In addition, cerebrospinal fluid (CSF) analysis revealed values within normal reference ranges, including CSF lactate 1.7 mmol/L, protein 0.22 g/L, glucose 3.6 mmol/L, and white blood cell count of 03 cells/mm^3^. Repeated chest x-ray showed substantial pulmonary interstitial congestion and an enlarged cardiac silhouette (Fig. 1B). The patient was managed with intravenous lidocaine, electrical cardioversion, and vasopressors (adrenalin and noradrenalin). However, she showed a poor response to these interventions, and an additional ECG revealed a discrete ventricular heart rhythm, followed by asystole (cardiac arrest). She was further managed with chest compression, continued lidocaine administration, and increased vasopressor doses. Since the patient showed minimal improvement with intensive treatments and CRRT, she was indicated to undergo extracorporeal membrane oxygenation (VA-ECMO) in combination with the CRRT. Simultaneously, a pacemaker was set up for this patient because of life-threatening ventricular arrhythmia refractory to intravenous lidocaine.

**Table 1:** Clinical features and outcome of the patient with severe HFMD by Enterovirus A71 managed with combined VA-ECMO and CRRT.

Age, sex, comorbidity	Clinical status on PICU admission	Clinical status at time point of commencing ECMO	Radiological findings	Clinical status and complications after experiencing ECMO	Treatments	Outcomes`
5-year-old, female No underlying disease on admission	High fever, substantially vomiting, lethargy and inactive, markedly startling jerks, and weakness of limbs, mouth ulcers, no vesicular rash, mottled skin, increased breath rate 70 per min, rapid weak pulse, sinus rhythm 198 bpm, blood pressure 128/78 mm Hg, CRT > 3s **Laboratory tests:** WBC = 18 × 10^9^/L Hb = 14.1 g/dL PLT = 446 × 10^9^/L Procalcitonin = 0.9 ng/mL Blood lactate = 5.8 mmol/L Creatinin = 47 µmol/L Troponin I = 7.36 ng/L AST/ALT = 49/15 U/L **Arterial blood gases:** pH = 7.38 pCO2 = 11 mm Hg BE = - 18.4 HCO3^-^ = 6.2 mEq/L On quick test: positive IgM Enterovirus A71	Patient was on mechanical ventilation and CRRT. Pulse 200 bpm, blood pressure 70/35, CRT > 3 s, response to pain, pupillary diameter = 2 mm and positive light reflex, muscle tones of lower limbs 5/5, right arm 3/5 and left arm 5/5 **Laboratory tests:** Blood lactate > 13.4 mmol/L Creatinin = 45 µmol/L Troponin I > 50 ng/L **Arterial blood gases:** pH = 7.21 pCO2 = 21 mm Hg BE = −19.5 HCO3^-^ = 7.4 mEq/L **CSF analysis:** CSF lactate = 1.7 mmol/L Protein = 0.22 g/L Glucose = 3.6 mmol/L WBC count = 03 cells/mm^3^ **Point-of-care cardiac ultrasound:** Hypodynamic heart, EF 30% **ECG**: initially ventricular tachycardia, then discrete ventricular rymthm 40 bpm, followed by asystole (cardiac arrest).	**Chest x-ray:** Substantial pulmonary congestion (bilaterally), enlarged cardiac silhouette **MRI:** Nodular lesions in the posterior medulla oblongata bilaterally, mild dilatation of bilateral ventricles and enlargement of subarachnoid space	Markedly clinical improvement at 8 hours after ECMO Strong regular pulse 150 bpm, blood pressure 105/76 mm Hg, CRT < 2s, urine output 2ml/kg/h Improved neurological status: eye opening on pain stimulation, limb movement, pupillary diameter = 2 mm and positive light reflex **Laboratory tests:** Blood lactate = 4.4 mmol/L Procalcitonin = 4.86 ng/mL Creatinin = 40 µmol/L Troponin I = 28 ng/L AST/ALT = 1545/437 U/L **Arterial blood gases:** pH = 7.4 pCO2 = 21 mm Hg BE = – 7 HCO3^-^ = 16 mEq/L PCR test confirmed Enterovirus A71, from rectal swabs **Complications:** 1. Right femoral artery rupture during catheterization procedure, managed with the left saphenous vein graft 2. Nosocomial infections detected by multiplex PCR from sputum samples revealed *E.coli, Stenotrophomonas maltophilia* and *Klebsiella.* spp pneumonia, despite negative results from conventional blood and CSF cultures.	AED, Chest compression, Mechanical ventilation, Intravenous immunoglobulin Lidocaine infusion, Vasopressors, Inotropes, IV antibiotics, CRRT VA-ECMO	Alive Discharged after 4 weeks in hospital, with mild weakness of right arm with muscle tone 1/5 One month after discharge, she showed markedly clinical improvement, muscle tone of her right arm increased by 4/5. Reduced size of the lesion in posterior medulla oblongata on MRI findings.

AED = automatic external defibrillator, BE = base excess, CRRT = continuous renal replacement therapy, CRT = capillary refill time, CSF = cerebral spinal fluid, ECG = electrocardiodiagram, EF = ejection fraction (%), MRI = magnetic resonance imaging, PCR = polymerase chain reaction, VA-ECMO = extracorporeal membrane oxygenation.

**Figure 3.**
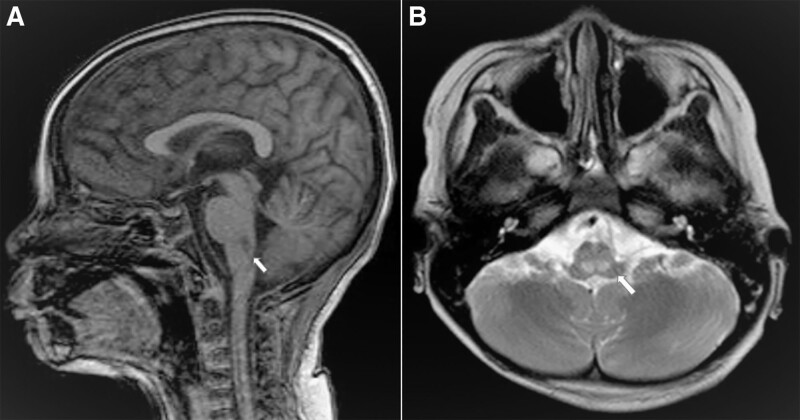
Magnetic resonance imaging (MRI) revealed small nodular lesions in the posterior medulla oblongata bilaterally, as indicated by the white arrows in the sagittal plane (A) and axial views (B).

**Figure 4.**
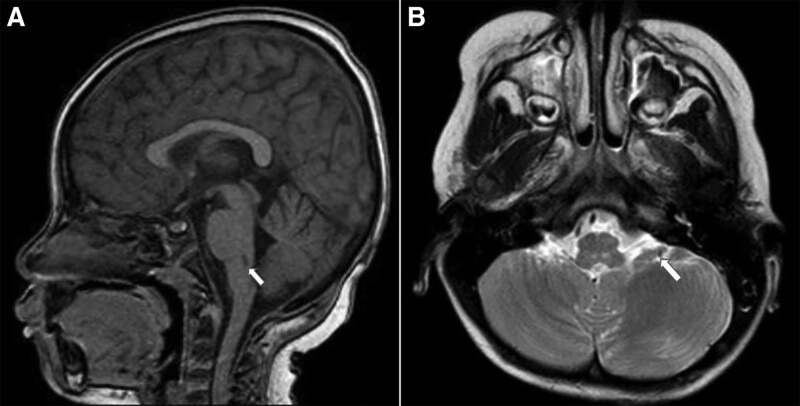
There was an improvement in MRI findings, with downsized nodular lesions in the posterior medulla oblongata bilaterally, as indicated by white arrows in the sagittal plane (A) and axial views (B).

Eight hours after undergoing VA-ECMO, there was significant clinical improvement; the patient presented with a regular sinus heart rhythm of 150 bpm, blood pressure of 105/76 mm Hg, CRT of < 2 seconds, urine output of 2 mL/kg/h. The neurological status improved, with eye opening to pain stimulus, a pupillary diameter of 2 mm, and a positive light reflex. Vasoinotropic doses were markedly tapered, and lidocaine infusion was continued at 20 µg/kg/min. Repeated laboratory tests revealed substantial improvement, as indicated by Troponin I (28 ng/L), serum lactate 4.4 mmol/L, and arterial blood gases (pH 7.40, pCO2 21 mm Hg, pO2 180 mm Hg, BE −7, and HCO3 16 mEq/L). However, markedly increased transaminase levels were observed, with an AST level of 1545 U/L and ALT level of 437 U/L. Repeated POCUS showed ameliorated cardiac function with EF 35% on day 1 and 48% on day 2 after VA-ECMO. On day 5 after VA-ECMO, she was fully alert, HR 140 bpm, BP 110/65 mm Hg, CRT < 2 seconds, urine output at 2.5 mL/kg/h, improved cardiac function (EF 52%), and chest radiography (Fig. 1C). Therefore, she was weaned off VA-ECMO, inotropes, and lidocaine. Monitored laboratory tests showed results within closely normal reference ranges, regarding serum lactate, Troponin I and liver enzymes. PCR testing of rectal swabs confirmed Enterovirus-A71. Notwithstanding, she further experienced high fever with increased levels of biomarkers, including C-reactive protein 40 mg/dL and procalcitonin 4.86 ng/mL, and negative blood and CSF cultures. However, PCR results from the blood and sputum samples revealed the presence of *E. Coli, Klebsiella* spp., and *Stenotrophomonas maltophilia*. Ventilated-associated pneumonia and PICU-associated sepsis were the most likely diagnoses. She was further treated with appropriate intravenous antibiotics and fully recovered. In addition, she experienced a significant complication of rupture in the right femoral artery during the catheterization procedure and underwent further management with the left saphenous vein graft. Approximately 1 week after being weaned from VA-ECMO, she was fully alert and able to walk independently, although the patient still had intermediate weakness in the right arm with a muscle tone of 2/5. Importantly, all laboratory tests returned to normal values. Radiologically, magnetic resonance imaging (MRI) of the brain revealed small nodular lesions in the posterior medulla oblongata bilaterally, mild dilatation of the bilateral ventricles, and enlargement of the subarachnoid space (Fig. 3). At week-4 after hospital admission, she was clinically stable and discharged, although weakness in the right arm was noticeable with a muscle tone of 1/5, while the remaining upper and lower limbs showed normal function. She was recommended physiotherapy. A reexamination 1 month after hospital discharge showed considerable clinical improvement, as she could use her right hand for drawing, and the muscle tone of the right arm improved to 4/5. Additionally, her brain MRI findings revealed marked improvement, as indicated by the reduced lesion size in the posterior medulla oblongata (Fig. 4).

### 2.1. Continuous renal replacement therapy

CRRT was performed using a Gambro Prismaflex® hemofiltration system (Baxter, Gambro Industries, France) and a polymembrane AN69 filter. Various subtypes of AN69 filters were used, with the HF20 filter for children weighing < 10 kg, M60 filter for children weighing 10 to 15 kg, and M100 filters for patients weighing > 15 kg.^[8]^ A Balton dialysis catheter (Balton, Warsaw, Poland) was inserted into the internal jugular or femoral vein. The size of the catheter was selected based on the patient’s weight. The filtration mode of Continuous venovenous hemodiafiltration (CVVHDF) combined with convection and dialysis was performed. The average infusion pump rate was 5 mL/kg/min. Dialysis was performed using Prismasol B0® (Gambro Dasco S.p.A., Sondalo, Italy). In cases in which the extracorporeal circulatory volume exceeded 15% of the blood volume, the priming procedure was manipulated by mixing red blood cells with normal saline 0.9% to obtain a hematocrit of 45%.

### 2.2. Extracorporeal life support protocol

The rotaflow centrifugal pump ECMO system and PMP Maquet Quadrox-ID oxygen exchange membrane with Bioline coating (Maquet Cardiopulmonary AG, Germany) were primed with 0.9% sodium chloride mixed with 10 IU/mL heparin. This system was then connected to an HLS Arterial Cannula 15Fr placed in the right internal jugular vein and a Bio-Medicus Arterial Cannula 12Fr (Medtronic, Inc.) was placed in the right common femoral artery (CFA). Additionally, we exposed the right superficial femoral artery and placed an arterial reperfusion line with a Radifocus Arterial Cannula 5Fr (Terrumo Corporation, Japan). The VA-ECMO system parameters were initially set at 2500 rotations per minute, blood flow of 1.5 L/min, gas blender adjusted to 1.5 L/min and FiO2 at 100%, and the system was further adjusted based on the patient’s conditions.^[9]^ ECMO circulation was warmed using a HU 35 Heater Unit system (Maquet Cardiopulmonary AG). Bedside ultrasound and X-rays were performed to ensure that all cannulae were in the appropriate positions. Furthermore, the patient’s right leg tissue perfusion was continuously monitored using an Invos 5100c system (Medtronic, Inc.). Circulatory volume deficit is unavoidable when connecting a high-volume ECMO system to young pediatric patients; therefore, we transfused packed red blood cells and albumin as soon as VA-ECMO was established to maintain an effective circulating volume for the patient.

## 3. Discussion

In this study, we present a pediatric patient with severe HFMD due to Enterovirus A71 who experienced cardiopulmonary failure complicated by refractory ventricular tachycardia and cardiac arrest. The patient was treated with CRRT and VA-ECMO, which led to a markedly favorable survival outcome.

Enterovirus A71-related cardiopulmonary failure is associated with high mortality rates in patients with severe HFMD. Recent studies have demonstrated that acute cardiopulmonary failure results from excessive pulmonary vascular permeability, increased sympathetic activity, and damage to cardiomyocytes due to the release of large amounts of catecholamine into the bloodstream.^[10]^ The systemic inflammatory response syndrome caused by the massive release of cytokines, known as “cytokine storm,” may contribute to increased vascular permeability. Other studies have also shown that significantly higher levels of interleukin (IL)-1β, IL-2, IL-6, IL-8, IL-10, IL-13, interferon-γ (IFN-γ), and tumor necrosis factor-α (TNF-α) are observed in patients with pulmonary edema than in those without pulmonary edema.^[11–13]^ In addition, cardiogenic toxicity is caused by the massive release of catecholamine (or catecholamine storm) resulting from brainstem inflammation. Consequently, this can lead to cardiac failure and cardiogenic shock.^[4]^ Zhang et al showed a significant elevation of substances from renin-angiotensin-system (RAS) and noradrenalin in children with moderate to severe HFMD compared to healthy control children.^[14]^ In addition, mouse experiments have revealed a correlation between RAS-associated chemicals, noradrenaline, and the severity of HFMD. High levels of these substances were also detected in the brains, muscles, and lungs of mice with severe HFMD.^[1]^ Based on these rationales, CRRT was administered to patients with severe HFMD. First, CRRT can primarily regulate the levels of inflammatory cytokines through mechanisms of convection and adsorption on the dialysis membrane from the circulation of patients with severe HFMD.^[15,16]^ Second, whether CRRT can significantly reduce catecholamine levels in patients with severe HFMD remains unclear and requires further investigation.^[6,17,18]^ Third, several case series have demonstrated that CRRT enhances the survival outcome of patients with severe HFMD (stages 3 and 4), with more favorable outcomes for stage 3 than for stage 4.^[4,6]^ Additionally, Wang et al reported a case series of 29 children with severe HFMD due to Enterovirus-A71, intervened with continuous veno-venous hemodiafiltration, which significantly reduced the plasma levels of catecholamine and RAS substances, and ameliorated cardiovascular functions.^[6]^ In-hospital mortality has improved by 17.6% in this patient cohort.^[6]^

To date, there has been no specific therapy for severe HFMD, particularly that caused by Enterovirus A71,^[2]^ and VA-ECMO is currently employed as a rescue therapy for patients experiencing cardiopulmonary failure and shows no response to conventional treatments. In 2012, Jan et al conducted a single-intervention ECMO study, saving the lives of 10/13 (77%) HFMD children with cardiopulmonary failure, and this cohort showed better neurological outcomes.^[3]^ Another study by Jan et al in 2021 involved 17 children with severe HFMD managed with VA-ECMO, and 14/17 (82%) patients survived, as compared to another historic cohort of 10 patients, in which only 3/10 (30%) survived without VA-ECMO interventions.^[4]^

Our patient presented with severe HFMD complicated by cardiopulmonary failure, lidocaine-resistant ventricular tachycardia, and cardiac arrest. This patient proved unresponsive to all conventional treatments recommended by the WHO 2011 guidelines,^[2]^ as well as CRRT. Consequently, VA-ECMO was considered the last rescue treatment. We opted for VA-ECMO combined with CRRT for this patient based on the understanding that acute HFMD is typically a transient condition that may be reversible. Therefore, maintaining hemodynamic stability and restoring cardiac function to improve end-organ perfusion are of utmost importance, while VA-ECMO aids in the recovery of both myocardial function and pulmonary edema, and CRRT may regulate cytokine and catecholamine levels.^[6]^ The patient’s clinical and laboratory parameters significantly improved 8h after VA-ECMO initiation. By day 5 post-VA-ECMO, cardiac function returned to normal, and CRRT and inotropes were successfully discontinued. It is worth mentioning that the time interval for cardiac function recovery and VA-ECMO commencement in the patient in this study was longer than that reported by Jan et al at 69h and 93h, respectively.^[3]^ The patient in this report experienced critical complications associated with extracorporeal procedures, including thrombosis, femoral artery ruptures requiring vascular reconstruction surgery, and nosocomial infections by *E. coli, Stenotrophomonas maltophilia*, and *Klebsiella spp*. pneumonia, and prolonged PICU stay. Nevertheless, this patient did not develop severe complications, such as critical multi-organ failure, in comparison to the patients reported by Jan et al.^[3]^

Notably, neurological sequelae associated with Enterovirus-A71 infection are of paramount importance among survivors. A meta-analysis showed that approximately 39% of children with stages 3 and 4 HFMD experienced critical long-term neurological sequelae, including limb weakness and atrophy, ventilator dependence, ataxia, and seizures.^[7]^ Notable issues include the inability to perform daily activities and delayed neurocognitive development, thus necessitating long-term medical and physical support for survivors.^[19]^ Injured sites in the brainstem that were attacked and destroyed by Enterovirus A71 will significantly determine unfavorable outcomes, including survival probability and the occurrence of neurological and cardiovascular complications. Based on the MRI findings, our patient was fortunate to have bilateral lesions in the posterior medulla oblongata. Injury to the pons juxtaposed with medulla oblongata could lead to fatality. Hence, assessing CNS and brainstem injuries at the time of commencing VA-ECMO can help predict the possibility of severe neurological sequelae in the future.

Timely intervention with extracorporeal membrane oxygenation in combination with CRRT is critical for improving clinical outcomes in children with severe HFMD due to Enterovirus A71. Jan et al stated that VA-ECMO should be indicated for HFMD patients experiencing refractory hypotension with poor blood perfusion to end organs.^[3]^ Based on current clinical and pathological studies, we advocate initiating CRRT alone as the first-line treatment rather than combined CRRT and VA-ECMO, which should initially be used for severe HFMD patients with cardiopulmonary failure. The combined intervention of VA-ECMO and CRRT should be reserved for cases in which CRRT alone proves ineffective. At our pediatric tertiary hospital, we considered VA-ECMO intervention for severe HFMD patients presenting with cardiopulmonary failure and consciousness level lower than pain (P) level on the AVPU scale or equivalent to a Glasgow Coma Scale from 8 to 11 points,^[20]^ accompanied by one of the following conditions: failure with CRRT alone within 6 to 8 hours after commencement, or circulatory collapse attributed to refractory cardiac arrhythmias.

This study highlights the clinical management and indications of combined CRRT and extracorporeal membrane oxygenation to enhance the survival outcome of severe Enterovirus A71-associated HFMD patients with cardiopulmonary failure, refractory arrhythmias, and cardiac arrest. Our study underscores the need for further prospective studies to investigate the effectiveness of combined treatments of VA-ECMO and CCRT in comparison to single interventions with either VA-ECMO or CRRT in improving clinical outcomes of patients with severe EV-A71-associated hand foot and mouth disease.

## Acknowledgments

We would like to express our sincere gratitude to the patient who willingly participated in this study and generously granted us permission to report their clinical details.

## Author contributions

**Conceptualization:** Thanh Tat Nguyen, Nguyen Trung Bao, Vo Thanh Luan, Trinh Huu Tung.

**Funding acquisition:** Do Chau Viet.

**Investigation:** Thanh Tat Nguyen, Nguyen Trung Bao, Vo Thanh Luan, Bui Thanh Liem, Vo Hoang Thien Nhu.

**Methodology:** Thanh Tat Nguyen, Vo Thanh Luan.

**Supervision:** Do Chau Viet, Trinh Huu Tung.

**Writing – original draft:** Thanh Tat Nguyen, Nguyen Trung Bao, Vo Thanh Luan.

**Writing – review & editing:** Thanh Tat Nguyen, Vo Thanh Luan, Do Chau Viet, Trinh Huu Tung, Sakib Burza.
